# Genome Sequence of a Novel Soil Actinomycete, *Protaetiibacter* sp. Strain SSC-01

**DOI:** 10.1128/MRA.01029-20

**Published:** 2021-02-04

**Authors:** Huiquan Duan, Chamil E. Fernando, Scott S. Crupper, Stephen D. Fields

**Affiliations:** a Department of Biological Sciences, Emporia State University, Emporia, Kansas, USA; Portland State University

## Abstract

The family *Microbacteriaceae* represents a diverse and important group of soil bacteria in the phylum *Actinobacteria*. Here, we report the genome sequence of a soil *Microbacteriaceae* strain, *Protaetiibacter* sp. strain SSC-01, the second putative species of the genus. Iron acquisition and xylose metabolism are central pathways identified in the annotated genome.

## ANNOUNCEMENT

The actinobacteria are dominant taxa in temperate soils and make significant contributions to processes such as polysaccharide digestion, antibiotic-based microbial inhibition, heavy metal chelation, and plant growth stimulation (1). The placement of many of the taxa within the phylum is in flux, and the *Microbacteriaceae* family has undergone numerous recent revisions with newly proposed genera (1–3).

The *Microbacteriaceae* strain SSC-01, which was collected from cultivated garden soil in east central Kansas, United States (38.42N, 95.85W), was isolated by passaging single colonies through multiple rounds of growth on Reasoner’s 2A agar (4) at 37°C. Other media supporting SSC-01 growth were blood agar (Difco BD, Sparks, MD, USA) with defibrinated sheep’s blood (Hemostat Labs, Dixon, CA, USA) and tryptic soy agar (Difco BD) supplemented with 10 μM FeCl_3_·6H_2_O. DNA was isolated from bacterial lawns grown on blood agar using the Quick-DNA fungal/bacterial miniprep kit (Zymo Research, Irvine, CA, USA). SeqMatic LLC (Fremont, CA, USA) prepared a genomic library with the TruSeq protocol targeting a 450-bp insert. Illumina MiSeq sequencing with a 500-cycle TruSeq kit (v.2 chemistry) generated 2,107,131 paired-end 251-bp reads. Adapters were removed with Cutadapt (v.2.5) (5), resulting in 1,057,609,266 bases of Illumina sequencing. A DNA library for Nanopore MinION sequencing, prepared with the ligation sequencing kit (SQK-LSK109; Oxford Nanopore Technologies, Oxford, UK), generated 24,000 reads averaging 4,285 bp. Illumina and Nanopore reads were uploaded to the public usegalaxy.org server (6) for processing with FASTQ Groomer (v.1.1.5), fastp (v.0.19.5+galaxy1), and/or Porechop (v.0.2.3) tools (7–9) using default settings. Unicycler (v.0.4.8.0) (10) was used to assemble 1.5 million paired Illumina reads (252-fold coverage) using 14,856 high-quality MinION reads (21.5-fold coverage) as a scaffold to generate a single 2,958,807-bp contig with a GC content of 71.5%. Circularity was confirmed by identifying Nanopore reads spanning the artificial ends of the assembled contig.

Annotation using the NCBI Prokaryotic Genome Annotation Pipeline (PGAP) server (11) identified 2,690 potential protein-coding genes, 52 RNA-coding genes, and 30 pseudogenes. Approximately 33% of the putative proteins have unknown function, but over 20 proteins are predicted to be involved in iron acquisition pathways, including siderophore-based import, hemolysin III toxins, and heme transporters. Hemicellulose metabolism (including xylan and xylose), terpene synthesis, and heavy metal chelation pathways each have 10 or more predicted enzymes, suggesting important roles for this microbe in plant-soil interactions (12, 13).

Needleman-Wunsch nucleotide alignments of full 16S rRNA gene sequences using NCBI default settings revealed the greatest identity to both *Lysinimonas* KACC 19322 (GenBank accession no. MT367295.1) and Protaetiibacter intestinalis (originally *Lysinimonas* sp. strain 2DFWR-13) (GenBank accession no. MH989600.1) at 98.6%. Global comparative analysis with the RAST SEED viewer (14) showed that the average identity of all orthologous protein sequences of SSC-01 is higher for *P. intestinalis* than for other closely related species (Table 1), leading to tentative identification of strain SSC-01 as a *Protaetiibacter* sp. This second putative species of the genus represents a different ecological niche than *P. intestinalis*, which was isolated from a larval *Protaetia* moth gut (3). A Mexico City, Mexico, landfill metagenome (GenBank accession no. RDDZ01000083.1), however, suggests the existence of other soil *Protaetiibacter* species.

**TABLE 1:** Species that are closely related to strain SSC-01 that have available complete genomic sequences^*a*^

Species/strain	GenBank accession no.	No. of orthologues shared with SSC-01^*b*^	Mean identity with SSC-001(%)
Leifsonia xyli CTCB07	NC_006087.1	1,581	55.6
*Lysinimonas* sp. KACC 19322	NZ_CP043504.1	2,129	71.9
*Lysinimonas* sp. SLBN-160	NZ_JACBYT010000001.1	2,057	55.9
*Protaetiibacter intestinalis*	NZ_CP032630.1	2,317	75.7

a Predicted orthologous proteins and their average percent identity with their SSC-1 counterparts were determined with the sequence-based comparative software of the RAST SEED browser (14).

*^b^* Amino acid identity of 30% used as the threshold for orthology.

### Data availability.

This bacterial genome sequence has been deposited in DDBJ/ENA/GenBank under the accession no. CP059987. Raw sequence data used for assembly have been deposited in DDBJ/ENA/GenBank under the accession no. PRJNA649951. The assembly described in this paper is the first version (GenBank accession no. CP059987.1).
